# Identification and characterization of microsatellite loci in two socially complex old world tropical babblers (Family Timaliidae)

**DOI:** 10.1186/s13104-015-1684-9

**Published:** 2015-11-24

**Authors:** Sara A. Kaiser, J. E. Danner, Laura Bergner, Robert C. Fleischer

**Affiliations:** Center for Conservation and Evolutionary Genetics, Smithsonian Conservation Biology Institute, National Zoological Park, Washington, DC 20013 USA

**Keywords:** Borneo, Chestnut-crested yuhina, Cooperative breeder, Grey-throated babbler, Old World tropics, Parentage, Microsatellite, *Stachyris nigriceps*, *Staphida everetti*

## Abstract

**Background:**

Although the highest diversity of birds occurs in tropical regions, little is known about the genetic mating systems of most tropical species. We describe microsatellite markers isolated in the chestnut-crested yuhina (*Staphida everetti*), endemic to the island of Borneo, and the grey-throated babbler (*Stachyris nigriceps*), widely distributed across Southeast Asia. Both species belong to the avian family Timaliidae and are highly social, putatively cooperatively breeding birds in which helpers attend the nests of members of their social group. We obtained DNA from individuals in social groups breeding in Kinabalu Park, Malaysian Borneo.

**Results:**

We used a shotgun sequencing approach and 454-technology to identify 36 microsatellite loci in the yuhina and 40 in the babbler. We tested 13 primer pairs in yuhinas and 20 in babblers and characterized eight polymorphic loci in 20 unrelated female yuhinas and 21 unrelated female babblers. Polymorphism at the yuhina loci ranged from 3 to 9 alleles, observed heterozygosities from 0.58 to 1.00, and expected heterozygosities from 0.64 to 0.81. Polymorphism at the babbler loci ranged from 3 to 12 alleles, observed heterozygosities from 0.14 to 0.90 and expected heterozygosities from 0.14 to 0.87. One locus in the yuhina deviated significantly from Hardy–Weinberg equilibrium. We detected nonrandom allele associations between two pairs of microsatellite loci in each species.

**Conclusions:**

Microsatellite markers will be used to describe the genetic mating system of these socially complex species and to measure genetic parentage and relatedness within social groups.

## Findings

Tropical regions support the highest diversity of bird species than any other region worldwide [1]. Yet, we know little about the social behavior and mating systems of tropical birds [2, 3], especially Old World tropical species [4]. The chestnut-crested yuhina (*Staphida everetti*) is an endemic, resident bird that lives in tropical montane forests on the island of Borneo [4, 5]. The grey-throated babbler (*Stachyris nigriceps*) is a common resident of the tropical submontane forests of Northeast Indian subcontinent, southern China, Southeast Asia and Sumatra [4]. Both species belong to the Old World avian family Timaliidae, which is compromised of oscine passerine birds generally known as babblers [6]. Babblers show striking diversity in their social behaviors and mating systems [5]. The chestnut-crested yuhina (hereafter, yuhina) is a highly social bird that forages throughout the canopy in large single-species flocks of 10–30 birds [4] and the grey-throated babbler (hereafter, babbler) forages in small groups of 5–8 individuals during the breeding months [4, 7]. Both species are putatively cooperative breeders in which helpers attend the nests of their social group members (T. E. Martin unpubl. data). We describe the isolation and characterization of eight polymorphic microsatellite loci in each species that we will use to measure genetic parentage and relatedness between breeders and their offspring and helpers and to investigate the social structure, dispersal, and genetic mating system of these species.

We used 454 GS-Junior shotgun sequencing to develop species-specific microsatellite markers. Genomic DNA was extracted from whole blood stored in lysis buffer [8] with the DNEasy Blood and Tissue DNA Kit (Qiagen, Valencia, CA). Genomic DNA (2 µg) was sheared on the Q800R sonicator (QSonica, Newton, CT) for 2 min into 300–500 bp fragments. The sheared DNA sample was purified with Sera-Mag Speed Beads (2×) (Thermo Fisher Scientific, Waltham, MA) and eluted in 15 μl of ddH_2_0. Purified samples were prepared for 454 sequencing using a shotgun library preparation protocol [9]. DNA fragments were blunt-ended and short adapters [9] ligated to the 3′ and 5′ ends of each fragment with the NEB Quick Blunting and Quick Ligase Kits (New England Biolabs, Ipswich, MA). One end of each fragment contained a unique sample-specific 8 bp barcode. Fragments with adapters successfully ligated were reamplified using emulsion PCR (emPCR) primers [9] and libraries were purified with Sera-Mag Speed Beads in PEG solution and size selected by gel extraction from a 1.5 % agarose gel with the MinElute Gel Extraction Kit (Qiagen). Libraries were quantified with the 454 Library Quantification Kit (Kapa). The yuhina single-stranded DNA (ssDNA) library had an average length of 500 bp and the babbler ssDNA library had an average length of 600 bp. We pooled the barcoded ssDNA libraries with one other individually barcoded species and conducted an emPCR at a concentration of 0.6 copies per bead with Lib-L Roche kits and reagents. The emPCR yielded 3 % enriched beads for sequencing a single PicoTiter plate on the 454 Genome Sequencer Junior System (GS-Junior, 454 Life Sciences, a Roche Company, Branford, CT). We used the Roche software shotgun pipeline for quality filtering on the GS-Junior, resulting in a total of 38,619 sequenced fragments for the three species. The 454 datasets were demultiplexed using a MIDconfig.parse file from the sfffile program. The single run yielded 17,645 reads for the yuhina (range 32–580 bp) and 8029 reads (range 32–548 bp) for the babbler, both with an average read length of 350 bp. We filtered the reads (min length = 60, max length = 400, ambiguity max 1 % of N, mean quality score = 15–25) and trimmed the run of low quality sequences (mean quality score: 5′ = 20, 3′ = 20) with PRINSEQ [10], resulting in 14,492 good reads for the yuhina and 6591 good reads for the babbler.

We used 454 sequence data to identify microsatellites and design PCR primer pairs. We screened for perfect and imperfect (>85 %) microsatellites (repeats of di-, tri- and tetranucleotides) with minimum repeat lengths of 20, 24, and 28 bp, respectively, with the Phobos plugin [11] in Geneious [12]. We selected unique repeat motifs with adequate length of flanking sequences (~25 bp) to design primer pairs (~18 bp) for amplifying these repeats. We used BLAST [13] to identify and remove microsatellite sequences that matched known bacteria over the length of the read. We aligned all reads with microsatellites to remove exact duplicates with Geneious. This resulted in 36 unique microsatellites in the yuhina and 40 in the babbler for primer design. We designed five primer pairs for each microsatellite locus with the Primer3 plugin [14] and chose the best pair of primers for each microsatellite based on a combination of least dimer (pair, self, and/or hairpin) and matching melting temperatures between primers with a size <300 bp.

Thirteen yuhina primer pairs and 20 babbler primer pairs were tested for amplification, optimized, and screened for polymorphism using DNA from unrelated females each from different social groups (yuhinas = 20 females, babblers = 21 females) sampled in Kinabalu Park, Malaysian Borneo. We amplified 1 μL of genomic DNA from each individual at each locus in a 10 μL PCR containing 4.15 μL dH_2_O, 1 μL 10× PCR buffer, 1.0 μL 25 mM MgCl_2_ (2.5 mM final concentration), 1.0 μL 10 mM deoxyribonucleotide triphosphates, 0.4 μL 10 μM forward and pigtail reverse primers, 1.0 μL of 2.5× bovine serum albumin, and 0.05 μL 5.0 U μL^−1^ AmpliTaq Gold DNA polymerase [Applied Biosystems (ABI), Carlsbad, CA]. We initially used touchdown cycling conditions decreasing by 0.5 °C for each cycle (55–65 °C for yuhinas and 50–60 °C for babblers) with unlabeled forward primers to test for amplification and to optimize the annealing temperatures (T_A_) for each primer pair. After determining the optimal range of T_A_, we added a 5′ fluorescent label (6-FAM, 5-HEX; Eurofins MWG Operon; NED, ABI) to the forward primer and a six base-pair ‘pigtail’ (GTTTCT) to the 5′ end of the reverse primer (babbler primers only) to promote adenylation of the 3′ end of the forward strand to improve genotyping accuracy [15]. We ran PCRs with fluorescently labeled forward primers on a DYAD thermal cycler (MJ Research) under the following conditions for yuhina primers: initial denaturing at 94 °C for 8 min, followed by eight cycles of 94 °C for 30 s, primer-specific upper T_A_ for 30 s and decreasing by 0.5 °C for each cycle, 72 °C for 1 min, followed by 35 cycles of 94 °C for 30 s, primer-specific lower T_A_ for 30 s, and 72 °C for 1 min, then a final extension at 72 °C for 30 min. We ran PCRs under the following conditions for babbler primers: initial denaturing at 94 °C for 8 min, followed by 45 cycles of 92 °C for 30 s, primer-specific T_A_ for 40 s, 72 °C for 40 s and a final extension at 72 °C for 7 min. The labeled PCR products were analyzed on an ABI PRISM 3130 Genetic Analyzer (ABI) and allele sizes were scored with the GeneScan 500 ROX size standard (ABI) in Genemapper v.4.1 (ABI). All primers amplified and eight of these primers were polymorphic in each species.

We characterized the eight polymorphic microsatellite loci for each species. We determined the number of alleles per locus (*K*) and calculated observed (*H*_*O*_) and expected heterozygosities (*H*_*E*_) (Table 2) with Arlequin v.3.5 [16]. The number of alleles per yuhina locus ranged from 3 to 9 (mean *K* ± SD: 6 ± 2) (Table 1). Observed heterozygosities ranged from 0.58 to 1.00 (mean *H*_*O*_ ± SD: 0.80 ± 0.14) and expected heterozygosities from 0.64 to 0.81 (mean *H*_*E*_ ± SD: 0.69 ± 0.06) (Table 1). The number of alleles per babbler locus ranged from 3 to 12 alleles (mean *K* ± SD: 6 ± 3) (Table 2). Observed heterozygosities ranged from 0.14 to 0.90 (mean *H*_*O*_ ± SD: 0.64 ± 0.24) and expected heterozygosities from 0.14 to 0.87 (mean *H*_*E*_ ± SD: 0.61 ± 0.23) (Table 2). We tested for deviations from Hardy–Weinberg equilibrium and for gametic disequilibrium with GenePop v.4.2 [17]. One yuhina locus deviated significantly from Hardy–Weinberg equilibrium (*StEv*114) even after Bonferroni correction. We found no evidence that the deviation from Hardy–Weinberg equilibrium was because of scoring error due to stuttering, allele dropout, or null alleles using Micro-Checker v.2.2.3 [18]. We detected nonrandom allele associations between two pairs of microsatellite loci in each species. We found gametic disequilibrium between *StEv*114 and *StEv*118 (Fisher’s exact test: *P* = 0.03) and *StEv*110 and *StEv*112 (Fisher’s exact test: *P* = 0.009). We found gametic disequilibrium between *StNi*102 and *StNi*105 (Fisher’s exact test: *P* = 0.01) and *StNi*105 and *StNi*114 (Fisher’s exact test: *P* = 0.03). The 16 microsatellite markers described here will be used to assess genetic parentage and relatedness within social groups and describe the genetic mating system of these socially complex species.

**Table 1:** Characteristics of eight microsatellite loci developed and optimized from the chestnut-crested yuhina, *Staphida everetti*

Locus	Repeat motif	Primer sequence (5′–3′)		T_A_ (°C)	Size range (bp)	*K*	*H*_*o*_	*H*_*E*_	HWE	GenBank accession no.
*StEv*26	(CA)_13_	F: AGCAATAGGACTGACACAAGGT		61–57	84–96	7	0.90	0.70	0.43	KT582127
		R: AGTCTTGATTTCCCCACTTTGTC								
*StEv*103*	(TAA)_15_	F: CCAGCCTCTGAACTGGTCTG		61–57	228–270	11	0.63	0.66	0.21	KT582120
		R: TTCTGTGGGTCTTGGTGGTT								
*StEv*106	(ATAG)_10_	F: TTGGACAAACAGCTGCTCCA		61–57	149–201	13	0.80	0.70	0.38	KT582121
		R: ACACACTTCAGCTGGAACTG								
*StEv*110*	(AAAT)_9_	F: TCCAGCATTTCTTCCTCTTGGA		62–58	181–199	9	0.58	0.66	0.11	KT582122
		R: AACAGATCCACACAGGCAGG								
*StEv*112	(TTGG)_11_	F: AGAAGCAGAGAGAGGTAGGAA		61–57	234–258	11	0.89	0.72	0.11	KT582123
		R: TGAGAAAGGATGCACGTGTTG								
*StEv*114*	(AT)_8_	F: TCCTTTTCTTTTCCTACTTTCATTTCT		61–57	164–180	6	1.00	0.64	<0.001	KT582124
		R: ACACAGCTTTGTGAGGGCTT								
*StEv*118	(GT)_13_	F: CACTGCCCAGTTTGGAATGC		61–57	120–152	11	0.84	0.81	0.35	KT582125
		R: CCAGCTCACACTCCTATGCC								
*StEv*122	(AC)_10_	F: AGTCCTCACTGTGCAGGTTG		61–57	244–252	5	0.74	0.64	0.13	KT582126
		R: ACATGCACACTGTGGACCAA								

*T*_*A*_ optimized touchdown upper and lower annealing temperatures, *K* number of alleles, *H*_*O*_, observed heterozygosity, *H*_*E*_ expected heterozygosity, *HWE* test for Hardy–Weinberg equilibrium

* Imperfect microsatellites

**Table 2:** Characteristics of eight microsatellite loci in the grey-throated babbler, *Stachyris nigriceps*

Locus	Repeat motif	Primer sequence (5′–3′)		T_A_ (°C)	Size range (bp)	K	H_o_	H_E_	HWE	GenBank accession no.
*StNi*102	(TG)_13_	F: TGAAGAATGTGGGTGGAGAAGT		58	171–179	5	0.71	0.65	1.00	KT592540
		R: GTTTCTTGCTTTAGATGGGCTCCTGC								
*StNi*104	(TAT)_16_	F: TCTGTCTGTGTTGGGGTTTATGT		58	148–194	15	0.81	0.82	0.53	KT592541
		R: GTTTCTGACCTCACATGCCAAGACCA								
*StNi*105*	(ACAG)_10_	F: TGGCAAACACACGTCAGTCT		58	156–188	8	0.57	0.55	0.47	KT592542
		R: GTTTCTCTCAACAAGGGCTCGAGGTT								
*StNi*107	(ATA)_14_	F: TCACATGTATAAGTTCCACAGTGA		58	179–250	21	0.90	0.87	0.56	KT592543
		R: GTTTCTACTCCAAACAGAACTACAGAGCT								
*StNi*111	(TCAA)_11_	F: AGCACGTTTACTCCAAACCA		58	208–200	4	0.80	0.70	0.36	KT592544
		R: GTTTCTGGTTTCAGCTTTGGTCCTTCC								
*StNi*112	(GTTT)_7_	F: TTTTGGAGGGTTGGCACAGT		58	211–237	12	0.67	0.67	0.81	KT592545
		R: GTTTCTTTGCCCAGTCCTTTGCTGAA								
*StNi*114*	(TTTG)_7_	F: AGAGGCTAGCTTGCTAAGGA		58	187–199	8	0.14	0.14	1.00	KT592546
		R: GTTTCTTGGTCTCATCAGTCGGCCTA								
*StNi*130	(TG)_10_	F: CTCTCCCTCCTCTCCGCC		58	200–246	8	0.53	0.50	0.71	KT592547
		R: GTTTCTTTTGCTGGAGCCTAAGACCC								

The pigtail sequence GTTTCT was added to the 5′ end of the reverse primer

*T*_*A*_ optimized annealing temperature, *K* number of alleles, *H*_*O*_ observed heterozygosity, *H*_*E*_ expected heterozygosity, *HWE* test for Hardy–Weinberg equilibrium

* Imperfect microsatellites

## Availability of supporting data

Microsatellite sequences were deposited in the National Center for Biotechnology Information (http://www.ncbi.nlm.nih.gov) and are accessible in GenBank (see Tables 1, 2 for the list of accession numbers).
